# Prion protein modulates endothelial to mesenchyme-like transition in trabecular meshwork cells: Implications for primary open angle glaucoma

**DOI:** 10.1038/s41598-019-49482-6

**Published:** 2019-09-11

**Authors:** Ajay Ashok, Min H. Kang, Aaron S. Wise, P. Pattabiraman, William M. Johnson, Michael Lonigro, Ranjana Ravikumar, Douglas J. Rhee, Neena Singh

**Affiliations:** 1 Department of Pathology, School of Medicine, Case Western Reserve University, Cleveland, Ohio 44106 USA; 2 Department of Ophthalmology, School of Medicine, Case Western Reserve University, Cleveland, Ohio 44106 USA; 3 Department of Ophthalmology, Duke University, Durham, NC USA

**Keywords:** Cell biology, Glaucoma

## Abstract

Endothelial-to-mesenchyme-like transition (Endo-MT) of trabecular meshwork (TM) cells is known to be associated with primary open angle glaucoma (POAG). Here, we investigated whether the prion protein (PrP^C^), a neuronal protein known to modulate epithelial-to-mesenchymal transition in a variety of cell types, is expressed in the TM, and plays a similar role at this site. Using a combination of primary human TM cells and human, bovine, and PrP-knock-out (PrP^−/−^) mouse models, we demonstrate that PrP^C^ is expressed in the TM of all three species, including endothelial cells lining the Schlemm’s canal. Silencing of PrP^C^ in primary human TM cells induces aggregation of β1-integrin and upregulation of α-smooth muscle actin, fibronectin, collagen 1A, vimentin, and laminin, suggestive of transition to a mesenchyme-like phenotype. Remarkably, intraocular pressure is significantly elevated in PrP^−/−^ mice relative to wild-type controls, suggesting reduced pliability of the extracellular matrix and increased resistance to aqueous outflow in the absence of PrP^C^. Since PrP^C^ is cleaved by members of the disintegrin and matrix-metalloprotease family that are increased in the aqueous humor of POAG arising from a variety of conditions, it is likely that concomitant cleavage of PrP^C^ exaggerates and confounds the pathology by inducing Endo-MT-like changes in the TM.

## Introduction

Prion protein (PrP^C^) is a cell surface glycoprotein known mostly for its obligate role in the pathogenesis of prion disorders, a group of neurodegenerative conditions characterized by extensive degeneration of the brain parenchyma and the neuroretina. The key pathogenic event in all prion disorders is a change in the conformation of PrP^C^ to a β-sheet rich PrP-scrapie (PrP^Sc^) isoform^1–3^. The resulting loss of function of PrP^C^ combined with gain of toxic function by PrP^Sc^ are believed to contribute to disease-associated pathology^4^. In support of the loss of function hypothesis, recent reports suggest that dysfunction of PrP^C^ impairs its ability to fine-tune the Ras homolog gene family member A (RhoA)-associated coiled-coil containing kinase (ROCK) signaling pathway, resulting in over-activation of ROCK and signaling through the LIMK-cofilin pathway^5,6^. Deletion of PrP in knock-out (PrP^−/−^) mice or silencing in neuronal cells produces a similar outcome, supporting a key role of PrP^C^ in regulating cytoskeletal homeostasis^7,8^. PrP^C^ also interacts with several extracellular matrix (ECM) proteins^9^, and its absence or altered function induces cell-ECM dyshomeostasis, resulting in loss of neuronal polarity and axonal degeneration in diseased brains^5,7,10^.

One of the principal outcomes of RhoA-ROCK activation is a shift from cell-cell interactions to cell-substrate interactions, a key event in PrP^C^-mediated epithelial to mesenchymal transition (EMT) in several cell types^8,11^. Trabecular meshwork cells also respond to RhoA-ROCK activation, and upregulate fibrillogenic proteins that deposit in the extracellular matrix (ECM) and increase its stiffness^12–14^. This compromises the response of ECM meshwork to fluctuations in intraocular pressure (IOP) and increases resistance to aqueous outflow, the hallmark of primary open angle glaucoma (POAG). Although TM cells do not show the phenotypic changes typical of EMT because of their endothelial nature, the endothelial to mesenchyme-like transition (Endo-MT-like) is sufficient to elevate the IOP and precipitate glaucoma^15^. It is encouraging to note that ROCK inhibitors have provided significant benefit in reversing this change, and are promising therapeutic agents for the management of POAG^16–18^.

While the emphasis on inhibitors of ROCK activation for the therapeutic management of POAG is well-placed, it is equally important to identify pathways upstream or parallel to RhoA-ROCK, and cross-talk between different pathways. Examples include signaling through integrins^19–21^, TGFβ2^21,22^, autotaxin-LPA^23^, JNK-paxillin^24^, and cross-talk between TGFβ, integrins, and the ECM^25^ to name a few. An additional possible player is PrP^C^, a documented inducer of EMT through β1-integrin and RhoA-ROCK activation^5,7,11,26^. This question deserves attention because PrP^C^ is cleaved by members of the disintegrin and matrix-metalloprotease family of enzymes that are upregulated in the aqueous humor (AH) of glaucomatous eyes^27,28^, and is likely to confound the pathogenesis and therapeutic management of POAG through cross-talk with other pathways^29^.

Here, we explored the expression and functional significance of PrP^C^ in the TM of human, bovine, and mouse eyes. We demonstrate that PrP^C^ is expressed in the TM of all three species, and plays a significant role in maintaining the ECM structure at this site. In addition, significant amounts of soluble PrP^C^ are present in the AH, suggesting constitutive or regulated shedding of membrane-bound PrP^C^ in the anterior segment^30^.

## Results

### Expression of PrP^C^ in human, bovine, and murine trabecular meshwork

**Figure 1:**
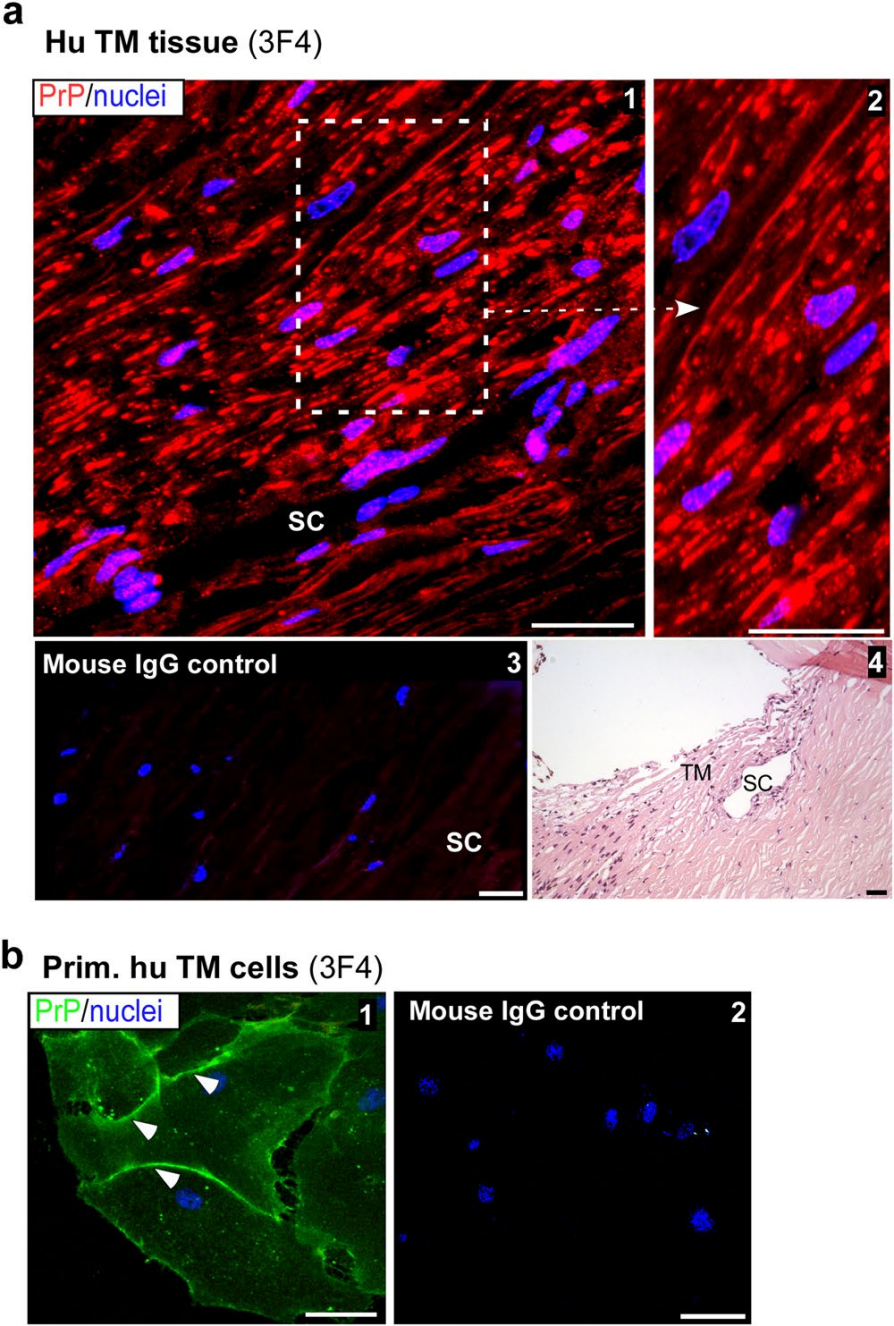
Distribution of PrP^C^ in the human trabecular meshwork. (**a**) Immunoreaction of human TM section with PrP-specific antibody 3F4 followed by Alexa fluor 546-conjugated secondary antibody shows strong reactivity in all layers of the TM (panel 1). High magnification image demonstrates expression of PrP^C^ on the plasma membrane of TM cells (panel 2). A serial section reacted with mouse IgG and Alexa fluor 546-conjugated secondary antibody shows no reaction (panel 3). H&E staining of a serial section confirms the TM region and Schlemm’s canal (SC) (panel 4). Scale bar: 25 µm. (**b**) Non-permeabilized primary human TM cells reacted with 3F4 followed by Alexa Fluor 488-conjugated secondary antibody show expression of PrP^C^ on the plasma membrane (panel 1). No reaction is detected in control cells exposed to mouse IgG followed by the same secondary antibody (panel 2). Scale bar: 25 µm.

Immunoreaction of human TM sections for PrP^C^ showed strong reaction in all layers of the TM (Fig. 1a, panels 1 & 2). Reaction with mouse IgG and H&E staining of serial sections confirmed the specificity of the immunoreaction and accurate identification of the TM region (Fig. 1a, panels 3 & 4). A similar evaluation of non-permeabilized primary human TM cells revealed expression of PrP^C^ on the plasma membrane as in neuronal and other cells (Fig. 1b, panel 1, arrowheads)^31^. No reaction was detected with non-specific mouse IgG processed in parallel (Fig. 1b, panel 2).

**Figure 2:**
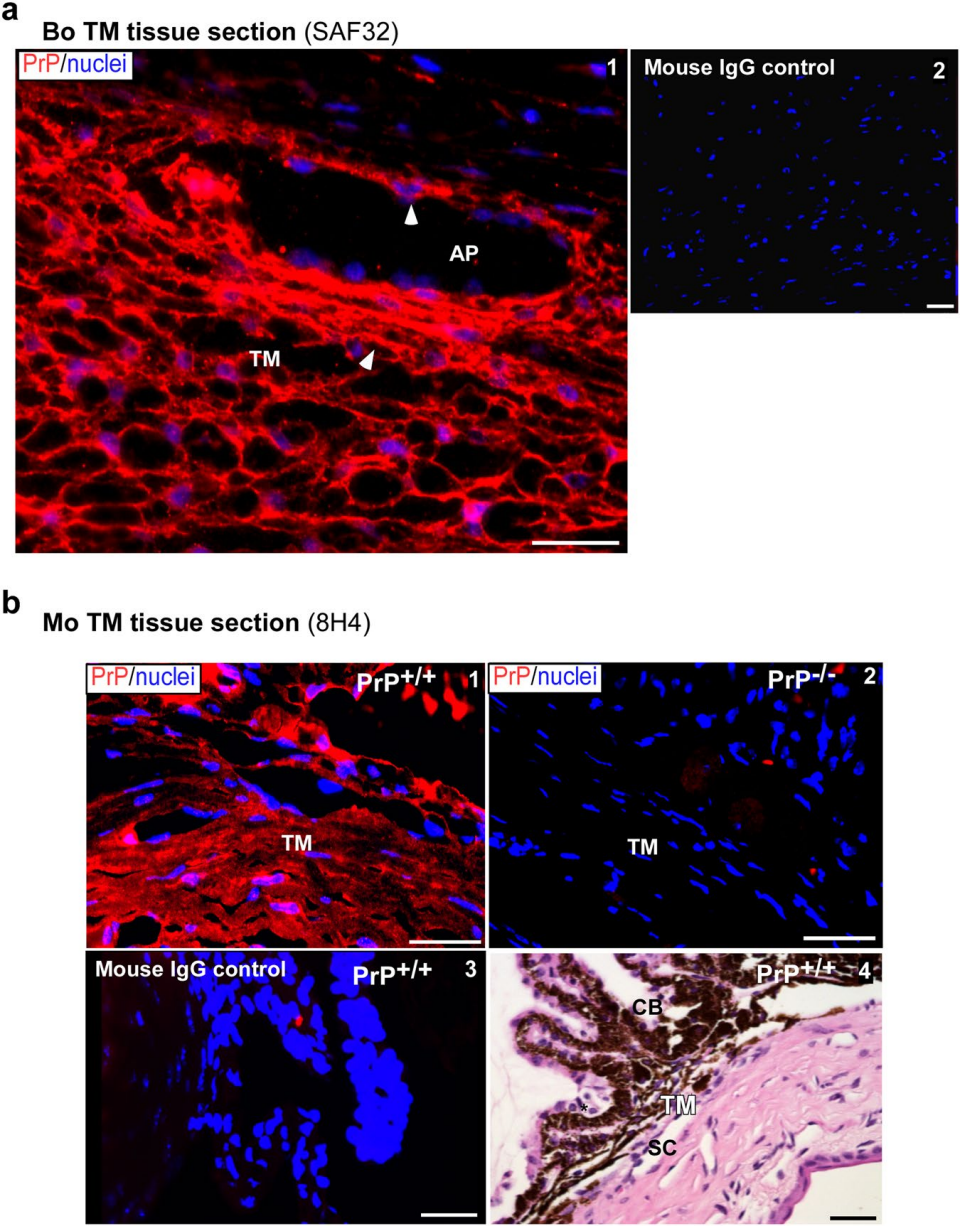
Expression of PrP^C^ in bovine and murine TM. (**a**) Immunoreaction of bovine TM section with PrP-specific antibody SAF32 followed by Alexa fluor 546-conjugated secondary antibody shows strong reactivity for PrP^C^ on the plasma membrane of TM cells and endothelial cells lining the aqueous plexus (AP) (panel 1, arrowhead). No reaction is detected in a serial section exposed to mouse IgG followed by the same secondary antibody (panel 2). Scale bar: 25 µm. (**b**) Immunoreaction of the anterior segment of PrP^+/+^ mouse eye with PrP-specific antibody 8H4 followed by Alexa fluor 546-conjugated secondary antibody shows expression of PrP^C^ in all layers of the TM (panel 1). No reaction is noted in PrP^−/−^ mouse sample processed in parallel (panel 2). Reaction of PrP^+/+^ sample with mouse IgG followed by the same secondary antibody shows no reaction (panel 3). H&E staining of a serial section confirms accurate identification of the TM region (panel 4). Scale bar: 25 µm.

Further confirmation of the above results was obtained by performing a similar analysis on bovine and mouse TM tissue (Fig. 2). Immunoreaction of fixed bovine TM sections for PrP^C^ revealed strong reactivity on the plasma membrane of TM cells and endothelial cells of the aqueous plexus (AP) (Fig. 2a panel 1, arrowheads)^32^. No reaction was detected in a serial section reacted with non-specific mouse IgG (Fig. 2a, panel 2). A similar evaluation of mouse TM sections showed strong reactivity for PrP^C^ in PrP^+/+^, and complete absence in PrP^−/−^ sections as expected (Fig. 2b, panels 1 & 2). Serial sections from the same block reacted with mouse IgG showed no reactivity (Fig. 2b panel 3). H&E staining confirmed accurate identification of the TM region in PrP^+/+^ sections of the anterior segment (Fig. 2b, panel 4).

### Processing of PrP^C^ in human ocular tissues

**Figure 3:**
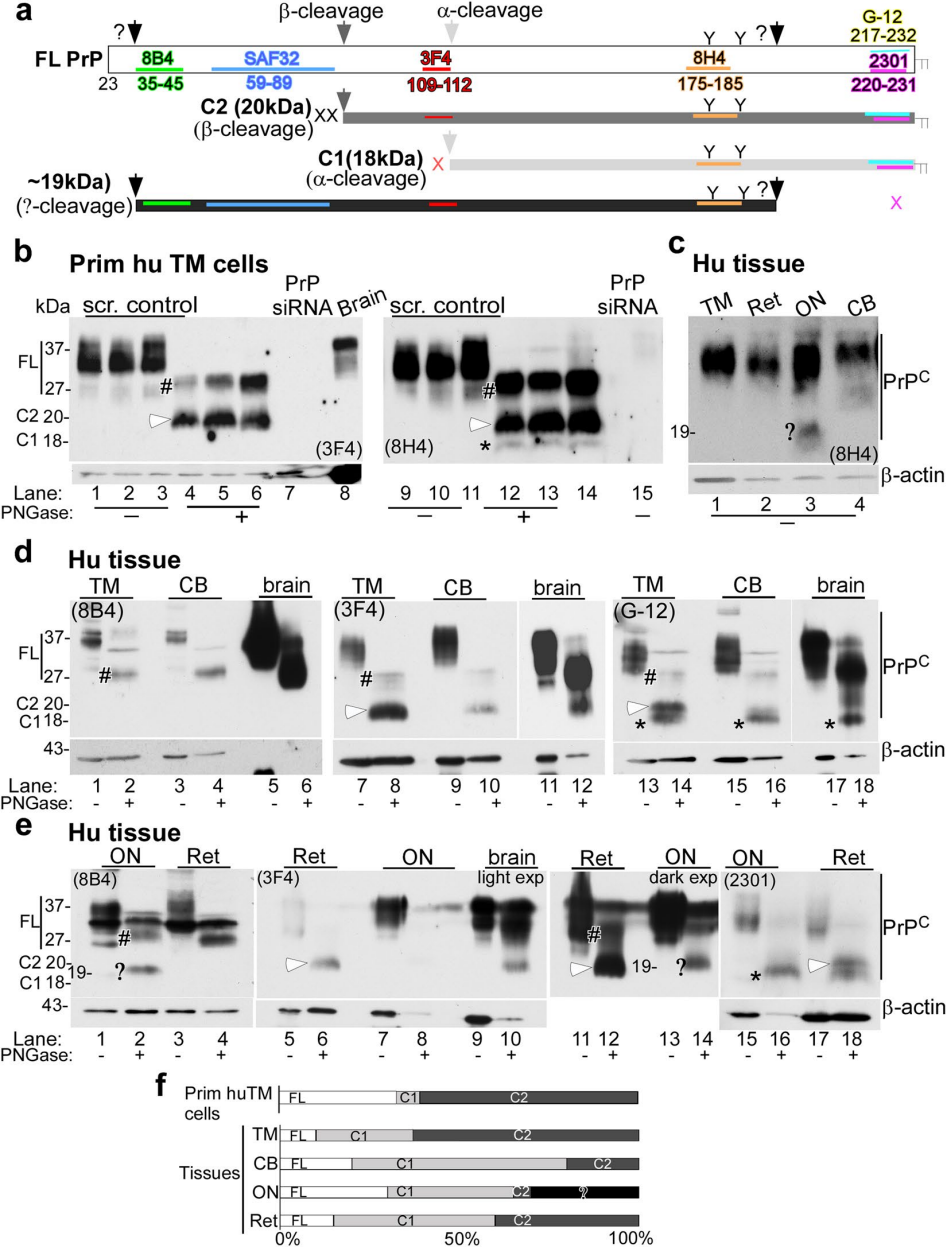
Processing of PrP^C^ in human ocular tissue. (**a**) Schematic representation of full length (FL), α-cleaved (C1, 18 kDa), β-cleaved (C2, 20 kDa), and ~19 kDa forms of PrP^C^. Antibody 8B4 reacts with FL and N-terminal fragments of PrP^C^, 3F4 reacts with FL and C2, and 8H4, G-12, and 2301 react with FL, C1, and C2. (**b**) Probing of lysates from primary human TM cells cultured from three different cases with 3F4 and 8H4 shows FL and mainly C2 fragment of PrP^C^. C1 represents a small fraction of total PrP^C^. Human brain lysate provides a positive control, and lysates from cells transfected with PrP-siRNA serve as a negative control (lanes 1–15). (**c**) Probing of lysates from the TM, retina (Ret), optic nerve (ON), and CB with 8H4 shows glycosylated PrP^C^ in all samples (lanes 1–4), and a ~19 kDa fragment in lysates from the ON (lane 3,?) (**d**) Probing of human TM and CB lysates with 8B4, 3F4, and G-12 shows FL glycosylated and deglycosylated PrP^C^ in all samples as in human brain. The TM shows significantly more C2 relative to FL and C1, the CB shows mainly C1, while the brain shows mainly FL and a small amount of C1 (lanes 1–18) (lighter exposures are shown for lanes 11 and 12. Complete membrane is shown in Supplementary Fig. S2). TM and CB lysates probed with 2301 antibody mimicked the G-12 probing data (Supplementary Fig. S3) (**e**) Probing of lysates from the ON and retina with 8B4, 3F4, and 2301 shows FL PrP^C^ as in human brain, and a ~19 kDa fragment in deglycosylated ON sample. The retina has significantly more C2 relative to FL and C1, while the ON has more C1 relative to C2. The ~19 kDa fragment does not react with 2301 (lanes 15-18). (FL: #; C1: star; C2: white arrowhead;?: ~19 kDa). All membranes were re-probed for β-actin to control for loading. (**f**) The relative abundance of FL, C1, C2, and the ~19 kDa fragment is shown graphically. Figures 3c-e are from tissue harvested from the same eye. Similar results were obtained from two other eye globes.

To evaluate the expression and processing of PrP^C^ in different regions of the eye, antibodies spanning the entire sequence of PrP^C^ were used (Fig. 3a). Thus, lysates of primary human TM cells were evaluated as such, or deglycosylated before processing for Western blotting. Probing of lysates with 3F4 that reacts with full-length (FL) and β-cleaved PrP^C^ showed glycosylated and unglycosylated forms as expected (Fig. 3b, lanes 1–3). However, deglycosylation with PNGase-F revealed that 30–50% of FL PrP^C^ (#, 27 kDa) was cleaved at the β-site (C2, white arrowhead, 20 kDa) (Fig. 3b, lanes 4–6; Fig. 3f). No reaction was detected in samples from TM cells transfected with PrP^C^-specific siRNA (Fig. 3b, lane 7). Lysate from human brain was fractionated in parallel as a positive control (Fig. 3b, lane 8). Re-probing with 8H4 showed α-cleaved 18 kDa band (C1, *), representing a minor fraction of the total (Fig. 3b, lanes 12–14). No reactivity was detected in samples treated with PrP^C^-specific siRNA (Fig. 3b, lane 15).

Evaluation of tissue from different regions of the human eye with 8H4 showed strong reactivity for FL PrP^C^ in the TM, retina (Ret), optic nerve (ON), and ciliary body (CB) (Fig. 3c, lanes 1–4, #)). However, the presence of a unique 19 kDa band in the ON was surprising (Fig. 3c, lane 3), prompting additional exploration.

To achieve this goal, three pairs of human eyes were dissected to isolate the TM, CB, retina, and ON, and tissue homogenates were evaluated as such or deglycosylated before Western blotting. Lysate from human brain was fractionated in parallel as a positive control. To avoid experimental artifacts, membranes probed once with a particular PrP^C^-specific antibody were re-probed only for β-actin, not with another PrP antibody.

Probing of lysates from the TM and CB with 8B4 that reacts with FL and N-terminal fragments revealed mainly FL PrP^C^ in all samples (Fig. 3d, lanes 1–6, #). N-terminal fragments of α- and β-cleaved PrP were not detected because these are soluble and unlikely to be present in significant amounts in tissue lysates^33^. Probing with 3F4 showed mainly β-cleaved PrP^C^ (Fig. 3d, lanes 7–10, white arrowhead). Reaction with G-12, a C-terminal antibody that reacts with FL, β-cleaved, α-cleaved, and probably Ɣ-cleaved soluble FL PrP^C^ showed mainly β-cleaved PrP^C^ in the TM, and α-cleaved fragment in the CB and brain samples (Fig. 3d, lanes 13–18, white arrowhead, *). Similar results were obtained with 2301, validating the results with G-12 (Supplementary Fig S3). In bovine CB, however, PrP^C^ showed equivalent representation of α- and β-cleaved forms^30^, suggesting species-specific differences in its proteolytic processing.

**Figure 4:**
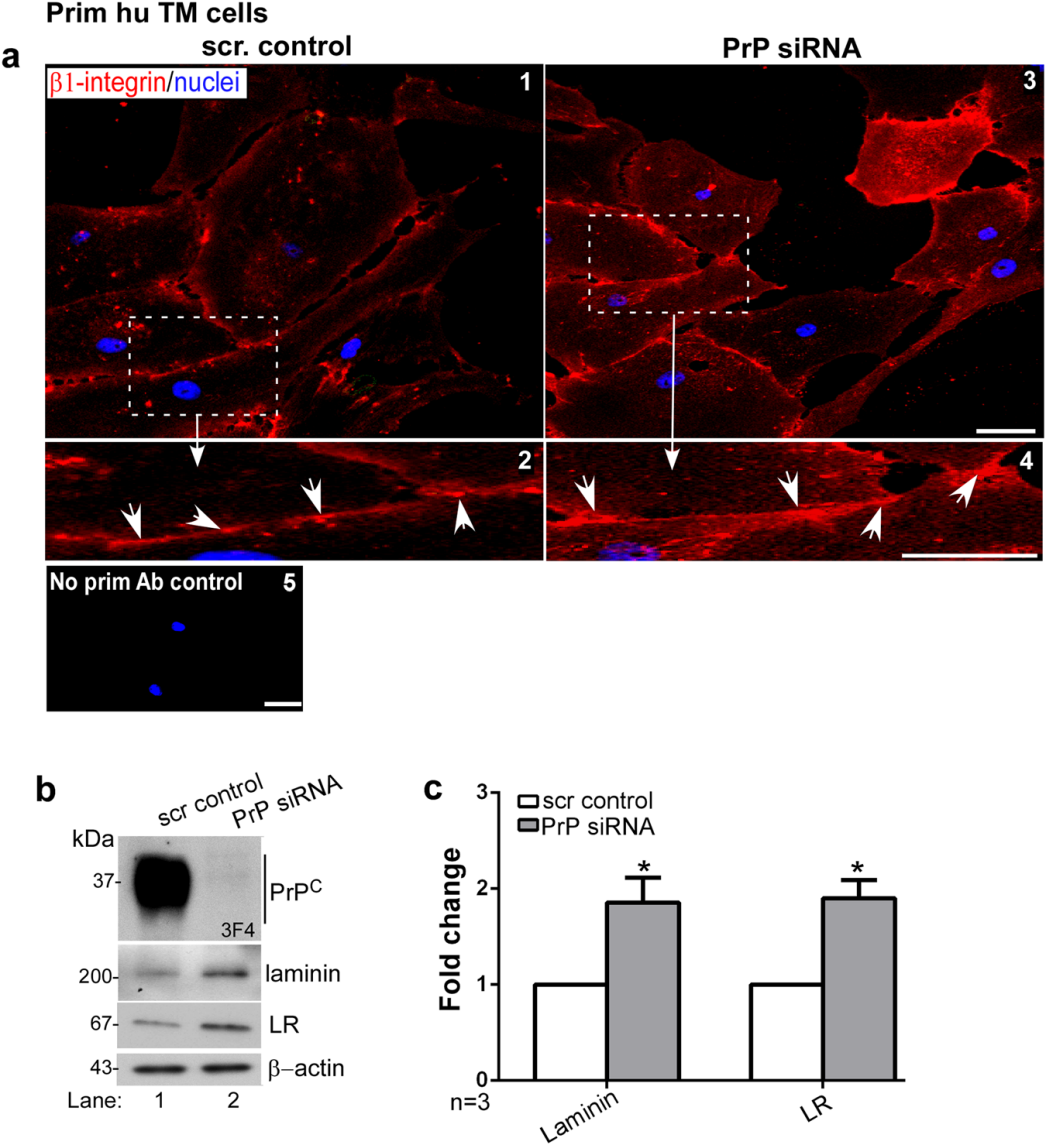
Downregulation of PrP^C^ aggregates β1-integrin and upregulates laminin and its receptor. (**a**) Silencing of PrP^C^ in human TM cells followed by immunostaining with antibody specific for activated β1-integrin shows clustering of activated β1-integrin on the plasma membrane in the absence of PrP^C^ (arrowheads) (panels 1–4). Cells transfected with PrP-siRNA and reacted with mouse IgG and respective secondary antibody do not show any reaction (panel 5). Scale bar: 25 µm. (**b**) Probing of TM cell lysates for PrP^C^ shows the expected glycoforms in control cells transfected with scrambled siRNA, and minimal reaction in cells exposed to PrP-siRNA (lanes 1 & 2). Probing for laminin and laminin receptor (LR) shows significant upregulation in the absence of PrP^C^ relative to control (lanes 1 & 2). (**c**) Quantification by densitometry after normalization with β-actin shows 2-fold upregulation of laminin and LR due to downregulation of PrP^C^. Values are mean ± SEM of the indicated n. *p < 0.05. Full-length blots are included in the Supplementary Fig. S2.

Lysates from ON and retina showed FL and α-cleaved PrP^C^ (Fig. 3e, lanes 1–18, #, *). The retina also showed β-cleaved PrP^C^ (Fig. 3e, lanes 6, 12, and 18, white arrowhead). Surprisingly, the optic nerve showed a unique fragment of ~19 kDa that reacted with the N-terminal antibody 8B4 (Fig. 3e, lane 2,?). This fragment also reacted with 8H4 (Fig. 3c, lane 3) and 3F4 (Fig. 3e, lane 14), but not with 2301 (Fig. 3e, lane 16). These observations suggest that this fragment is distinct from α, β, and Ɣ-cleaved PrP^C^ for the following reasons; (1) it is glycosylated and hence includes at least one N-glycan (amino acid 175) (Fig. 3e, lanes 1 & 2, lanes 13 & 14), (2) it reacts with 8B4, suggesting the presence of N-terminal amino acid 35 (Fig. 3a), (3) it does not react with 2301 (Fig. 3a), and (4) migrates at ~19 kDa, a molecular mass that is inconsistent with Ɣ-cleaved or FL PrP^C^. Further exploration is necessary to characterize this fragment fully and understand the conditions that precipitate its cleavage.

Quantification of relative abundance of FL, C1 (α-cleaved), and C2 (β-cleaved) forms of PrP^C^ in different tissues showed a ratio of 32:6:62 for TM cells, 9:27:64 for TM tissue, 20:60:20 for CB, 30:35:5:30 (unique) for optic nerve, and 14:45:41 for the retina (Fig. 3f). These ratios are an approximation at best because of the efficiency of reactivity of different antibodies. However, it is clear that unlike brain and neuronal cells where 60–70% of PrP^C^ is cleaved at the α-site, processing of PrP^C^ in ocular tissues is distinct, and is mainly at the β-site in human TM tissue.

### Silencing of PrP^C^ induces mesenchyme-like transition in the TM

Absence of PrP^C^ has been reported to induce aggregation of β1-integrin in neuronal cells^8^, triggering the Rho/ROCK pathway. To evaluate if a similar process occurs in the TM, primary human TM cells were transfected with PrP-specific siRNA, and non-permeabilized cells were immunoreacted with antibody specific for activated β1-integrin. Downregulation of PrP^C^ resulted in clustering of β1-integrin on the plasma membrane as opposed to control cells that showed uniform distribution (Fig. 4a, panels 1–4, arrowheads). Control cells where the primary antibody was omitted did not show any reaction (Fig. 4a, panel 5). Western blotting of lysates did not show a significant difference in β1-integrin expression levels as reported in a previous study^5^ (Supplementary Fig. S4).

To evaluate whether clustering of β1-integrin activates downstream pathways resulting in Endo-MT-like transition, primary human TM cells were transfected with PrP-specific siRNA to downregulate PrP^C^, and the expression and distribution of fibrillogenic proteins indicative of Endo-MT-like transition was evaluated by Western blotting.

Probing for PrP^C^ revealed the expected glycoforms in cells transfected with scrambled siRNA, and >98% downregulation by PrP-specific siRNA (Fig. 4b, lanes 1 & 2). Probing for laminin and laminin receptor (LR) showed significant upregulation in the absence of PrP^C^ relative to controls (Fig. 4b, lanes 1 & 2; Fig. 4c). Immunoreaction of control and experimental cells for laminin and laminin-receptor confirmed the immunoblotting results (Supplementary Fig. S5).

**Figure 5:**
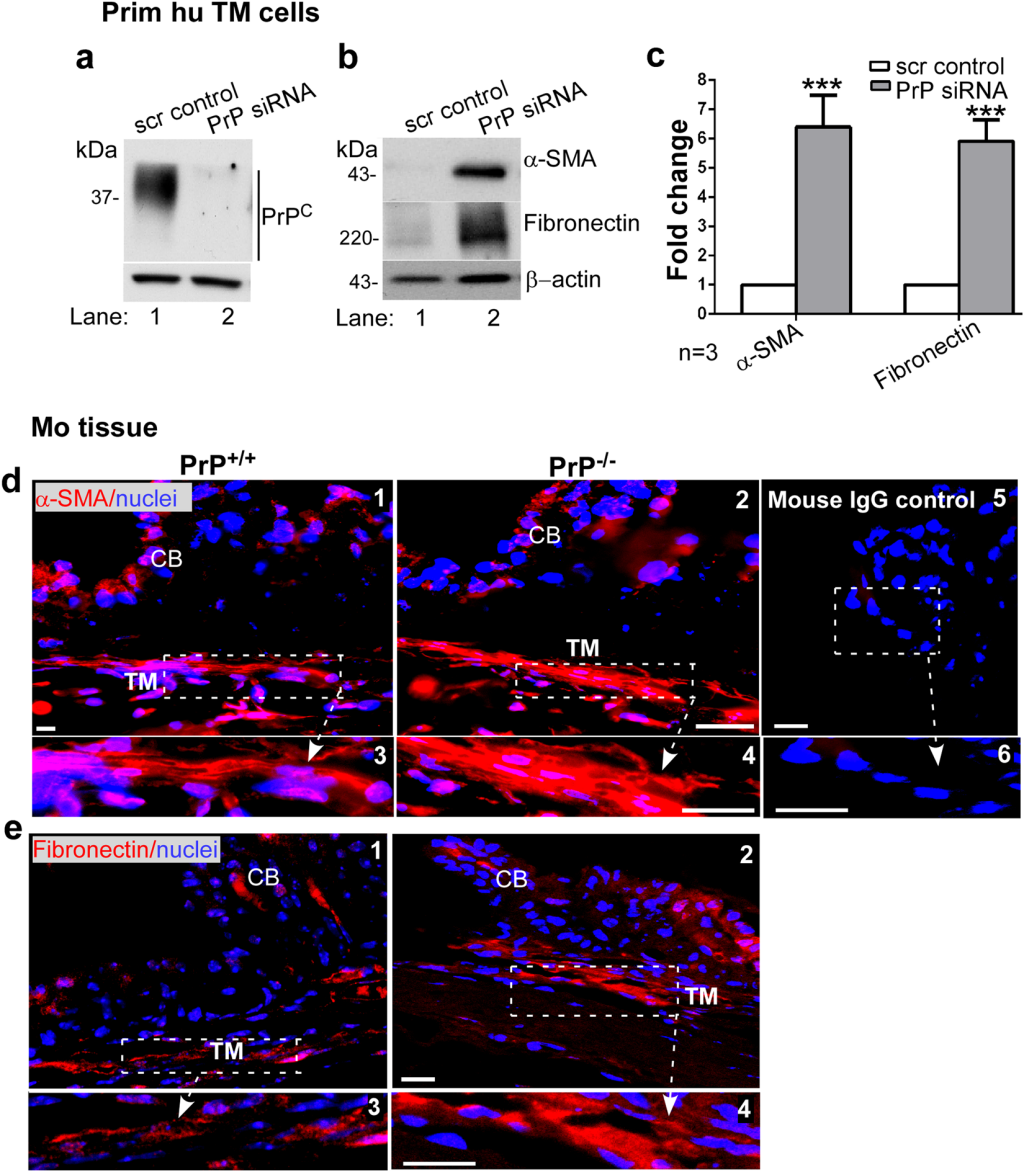
Downregulation of PrP^C^ upregulates α-SMA and fibronectin in the TM. (**a**) Probing of TM cell lysates treated with scrambled and PrP-siRNA for PrP^C^ shows the expected glycoforms in the scrambled control, and minimal reactivity for PrP^C^ in the experimental sample (lanes 1 & 2). (**b**) Probing of the same lysates for α-SMA and fibronectin shows upregulation in the absence of PrP^C^ relative to controls (lanes 1 & 2). (**c**) Quantification of protein expression by densitometry after normalization with β-actin shows 6.1-fold and 5.9-fold upregulation of α-SMA and fibronectin respectively. Values are mean ± SEM of the indicated n. *p < 0.05, **p < 0.01. Full-length blots (Supplementary Fig. S2). (**d & e**) Immunoreaction of fixed sections from the anterior segment of PrP^+/+^ and PrP^−/−^ mice shows stronger reactivity for α-SMA and fibronectin in the TM of PrP^−/−^ relative to PrP^+/+^ samples (panel 1 vs. 2). Scale bar: 25 µm. No reaction was detected in samples reacted with mouse IgG followed by Alexa 546-conjugated secondary antibody (panels 5 & 6).

A similar evaluation of duplicate cultures showed significant downregulation of PrP^C^ by siRNA treatment as above (Fig. 5a, lanes 1 & 2). Notably, downregulation of PrP^C^ resulted in significant upregulation of α-smooth muscle actin (α-SMA) and fibronectin relative to controls (Fig. 5b, lanes 1 & 2; Fig. 5c). Immunostaining of sections from PrP^−/−^ and PrP^+/+^ mouse eyes for α-SMA and fibronectin showed more reaction in the TM of PrP^−/−^ relative to PrP^+/+^ controls (Fig. 5d & 5e, panels 1–4), supporting the above results. Immunoreaction with mouse IgG showed no reaction (Fig. 5d, panels 5 & 6).

**Figure 6:**
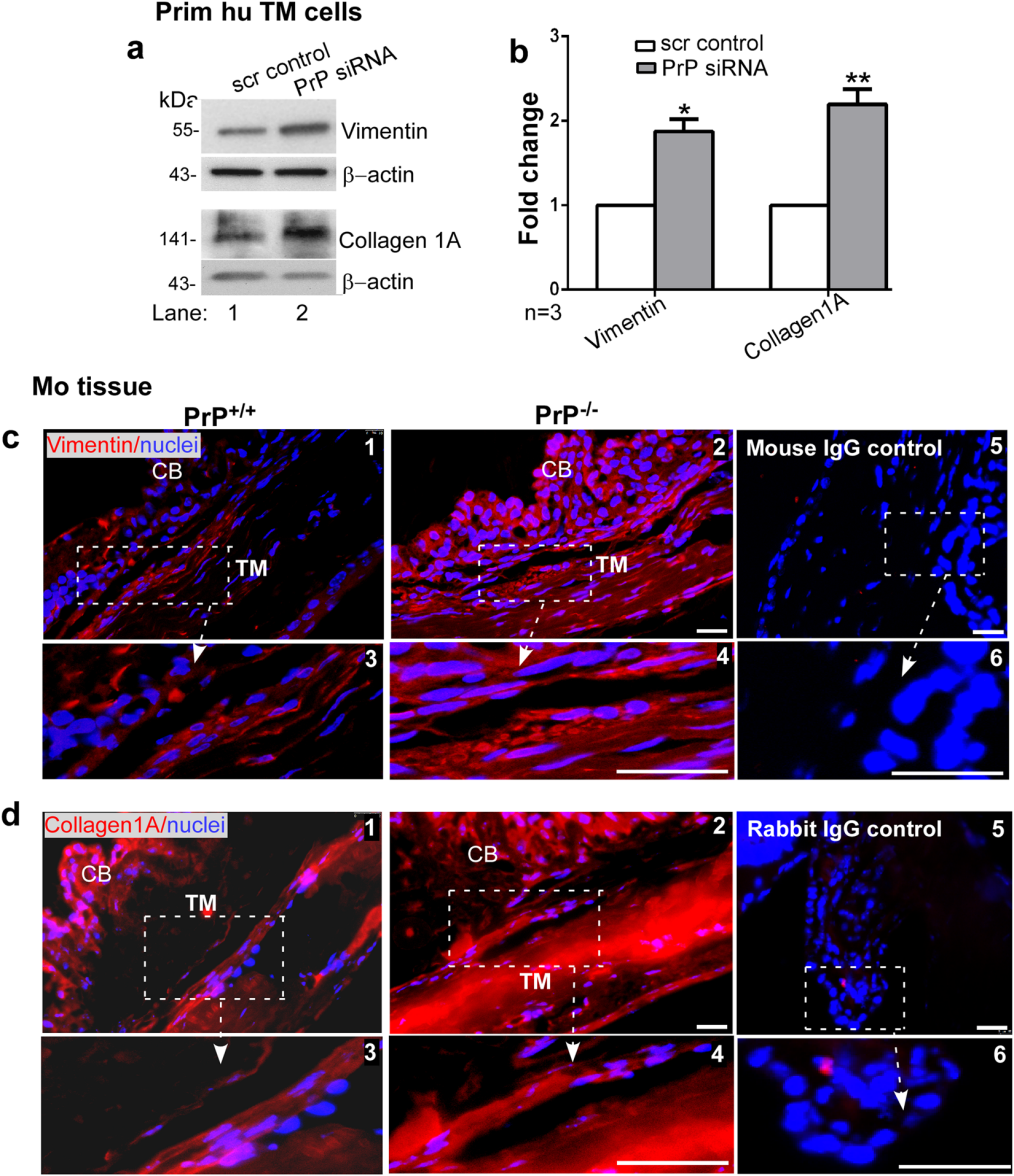
Downregulation of PrP^C^ upregulates vimentin and collagen 1A. (**a**) PrP^C^ was silenced in human TM cells and lysates were processed as above. Probing for vimentin and collagen 1A shows significant upregulation in the absence of PrP^C^ relative to controls (lanes 1 & 2). (**b**) Quantification of protein expression by densitometry after normalization with β-actin shows 1.9-fold upregulation of vimentin and 2.2-fold upregulation of collagen 1A in the absence of PrP^C^. Values are mean ± SEM of the indicated n. *p < 0.05, **p < 0.01. Full-length blots (Supplementary Fig. S2). (**c & d**) Immunoreaction of fixed sections from the anterior segment of PrP^+/+^ and PrP^−/−^ mice for vimentin and collagen 1A shows stronger reaction in the TM of PrP^−/−^ relative to PrP^+/+^ samples (panel 1 vs. 2). No reaction was detected when serial sections were reacted with mouse or rabbit IgG followed by Alexa 546-conjugated secondary antibody (panels 5 & 6). Scale bar: 25 µm.

Further probing of lysates from human TM cells transfected with scrambled and PrP-siRNA showed significant upregulation of vimentin and collagen 1A by downregulation of PrP^C^ in comparison to controls (Fig. 6a, lanes 1 & 2; Fig. 6b). Immunostaining of sections from the anterior segment of PrP^−/−^ and PrP^+/+^ mouse eyes for vimentin and collagen 1A showed more reactivity in PrP^−/−^ relative to PrP^+/+^ samples (Fig. 6c,d, panels 1–4), supporting the results from primary human TM cells. Immunoreaction with mouse IgG (for vimentin) and rabbit IgG (for collagen 1A) showed no reaction (Fig. 6c,d, panels 5 & 6).

Together, the above results demonstrate that absence or downregulation of PrP^C^ in mouse models or in primary human TM cells aggregates β1-integrin and upregulates fibrillogenic proteins including α-SMA, fibronectin, vimentin, and collagen 1A, the ECM protein laminin, and surprisingly, also the receptor for laminin.

### Absence of PrP^C^ upregulates myocilin

**Figure 7:**
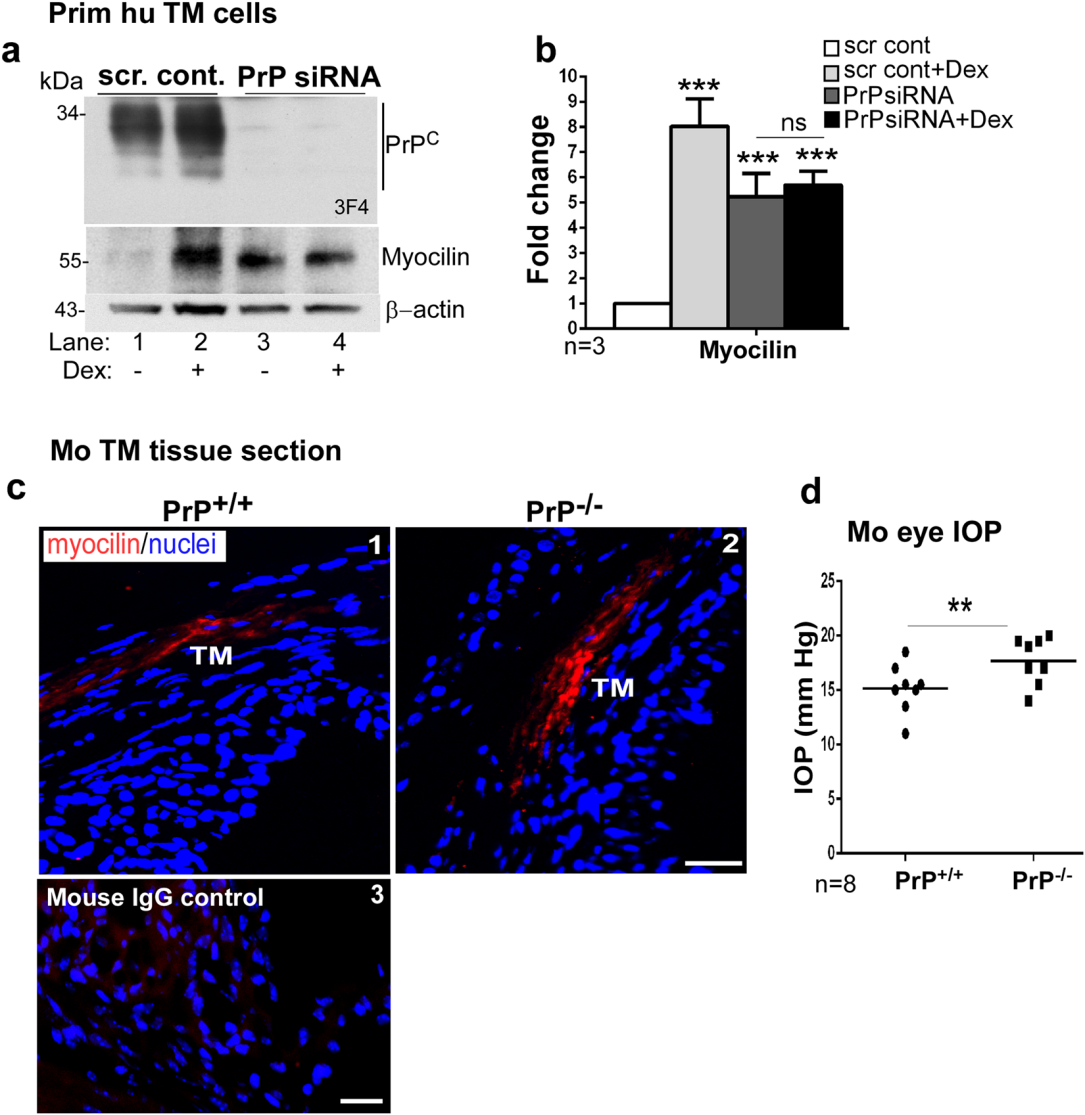
Myocilin is upregulated by downregulation of PrP^C^. (**a**) PrP^C^ was silenced in primary human TM cells and lysates were processed as above. Probing for PrP^C^ shows the expected glycoforms in controls, and minimal reaction in samples treated with PrP-siRNA (lanes 1 & 2 vs. 3 & 4). Re-probing for myocilin shows significant upregulation in the absence of PrP^C^ (lane 1 vs 3). Exposure of control and experimental cells to dexamethasone (Dex) shows upregulation of myocilin as expected (lanes 1 & 2). However, no additive effect of dexamethasone is noted in the absence of PrP^C^ (lanes 3 & 4). (**b**) Quantification by densitometry after normalization with β-actin shows 8-fold upregulation of myocilin by dexamethasone, and ~5.3-fold upregulation in the absence of PrP^C^ regardless of dexamethasone. Values are mean ± SEM of the indicated n. **p < 0.01; ns: not significant. Full-length blots are included in the Supplementary Fig. S2. (**c**) Immunoreaction of anterior segment of PrP^+/+^ and PrP^−/−^ mice for myocilin shows upregulation in the TM of PrP^−/−^ sections relative to controls (panels 1 & 2). No reaction was detected in a serial section reacted with mouse IgG followed by Alexa 546-conjugated secondary antibody (panel 3). Scale bar: 25 µm. (**d**) Measurement of IOP shows significant upregulation in PrP^−/−^ eyes relative to PrP^+/+^ controls (n = 8). Values are mean ± SEM of the indicated n. **p < 0.01.

Myocilin is a biomarker for TM cells, and is upregulated by dexamethasone treatment^34^. Since human TM cells are likely to change their morphology in culture, dexamethasone-mediated upregulation of myocilin, a reliable method for their validation^35^, was used before every experiment (Fig. 7a, lanes 1 & 2; Fig. 7b). Surprisingly, downregulation of PrP^C^ also upregulated myocilin in primary human TM cells (Fig. 7a, lane 1 vs. 3; Fig. 7b), and blunted their response to dexamethasone (Fig. 7a, lane 1 vs 3 & 4; Fig. 7b). Probing for PrP^C^ showed the expected glycoforms in cells treated with scrambled siRNA as expected, and almost complete absence in cells transfected with PrP-siRNA (Fig. 7a, lanes 1–4).

Immunoreaction of mouse TM sections for myocilin revealed significantly stronger reactivity in PrP^−/−^ samples relative to PrP^+/+^ controls (Fig. 7c, panels 1 & 2), confirming the results from human TM cells. Immunoreaction with mouse IgG showed no reactivity (Fig. 7c panel 3).

### Absence of PrP^C^ elevates intraocular pressure

To establish the clinical relevance of our observations to POAG, age and sex-matched PrP^+/+^ and PrP^−/−^ mice were anesthetized, and IOP was measured in both eyes with a tonometer. To eliminate bias, measurements were performed in separate sets of mice by three different individuals blinded to the mouse genotype. Surprisingly, IOP was significantly elevated in PrP^−/−^ eyes relative to PrP^+/+^ controls (Fig. 7d). The increase in IOP in PrP^−/−^ mice is in good agreement with upregulation of fibrillogenic proteins^36,37^, the hallmark of altered cell-ECM interactions in the TM.

## Discussion

We report that PrP^C^ is expressed in the TM, and modulates cell-ECM interactions at this site. Downregulation of PrP^C^ in primary human TM cells induced aggregation of β1-integrin on the plasma membrane and upregulation of fibrillogenic proteins. Likewise, fibrillogenic proteins were upregulated in the TM of PrP^−/−^ mice, indicating an Endo-MT-like transition. *In vivo* measurement of IOP revealed significant elevation in PrP^−/−^ relative to PrP^+/+^ mouse eyes, implicating PrP^C^ in the pathophysiology of POAG.

Our data demonstrate expression of PrP^C^ in the TM of human, bovine and mouse eyes, including endothelial cells of the Schlemm’s canal and the aqueous plexus (in bovine) that modulate aqueous outflow^12,38^. In primary human TM cells, PrP^C^ was detected on the plasma membrane as in neuronal and other cell types. However, unlike neurons where majority of PrP^C^ is cleaved at the α-site, most of the PrP^C^ in TM cells was cleaved at the β-site. Unlike α-cleavage that occurs during physiological recycling of PrP^C^ from the plasma membrane^39^, β-cleavage is associated with oxidative stress^40–42^, iron transport^30,43,44^, conversion of PrP^C^ to PrP^Sc^^1,3^, and possibly other stimuli^45^. It is surprising that human TM, ciliary body, optic nerve, and the retina showed distinct cleavage patterns of PrP^C^. In TM cells, TM tissue, and ciliary body, PrP^C^ was mostly β-cleaved, while in the retina PrP^C^ showed almost equal representation of α- and β-cleaved forms. Full-length PrP^C^ was minimal in all of the above ocular tissues. These observations differ from ~50% β-cleavage of PrP^C^ human retinal pigment epithelial cells^44^, and almost equal representation of α- and β-cleaved PrP^C^ forms in bovine ciliary body^30^. Since bovine and human eyes have different concentrations of oxalate, apo-transferrin, and possibly other anti-oxidants that determine susceptibility to light-induced oxidative stress^46^, it is likely that cleavage of PrP^C^ partly depends on the exposure of a particular ocular region to light or other stimuli that increase oxidative stress. It is notable that the optic nerve showed a novel internal fragment of ~19 kDa that requires further characterization. The soluble N-terminal fragments of α-, β-, and other cleaved forms of PrP^C^ are likely to accumulate in the AH and vitreous humor and play distinct physiological roles as in neurons^45^, a possibility that is currently under investigation.

The stimuli and the identity of enzymes responsible for the mainly β-cleavage of PrP^C^ in most ocular tissues and a unique cleavage in the optic nerve is not clear from our data. These are important unanswered questions with significant physiological and pathological implications^45,47–49^. In neuronal cells, PrP^C^ undergoes at least four different proteolytic events. α-Cleavage is predominant, and the neuroprotective role of the resultant N-terminal fragment N1 has been described^45^. The proteases responsible for this cleavage, however, are not clear, and are arbitrarily termed α-PrPases^48,50^. Cleavage near the C-terminus releases almost full-length PrP^C^ in the extracellular milieu, and is believed to protect neurons by reducing the substrate for PrP-scrapie, the disease-associated isoform of PrP^C^, on neuronal cells. Implications of soluble PrP^C^ in the extracellular milieu, however, are not clear. This cleavage is mediated by the disintegrin and metalloprotease ADAM10^51–53^. ADAM9 influences ADAM10 activity, and is thus indirectly responsible for this event^41,42,54^. Additional cleavage of mainly unglycosylated PrP^C^ near the C-terminus has been described, and is termed Ɣ-cleavage. The responsible protease is probably a member of the matrix metalloprotease family^42^. It is pertinent to mention here that matrix metalloproteases 2 and 9, ADAM proteases 9 and 10, and tetraspanin 6, a member of the tetraspanin family necessary for the maturation and transport of ADAM10, are increased in the AH of glaucomatous eyes of diverse etiology^27,55,56^. This raises the possibility that shedding of PrP^C^ from TM cells may induce Endo-MT-like transition and altered TM-ECM interactions, contributing to the ongoing pathology. β-cleavage of PrP^C^ is mainly associated with pathological conditions, and is mediated by calpains, lysosomal proteases, and oxidative stress. It is believed that the released N-terminal fragment N2 is an anti-oxidant and thus neuroprotective^45,49,57^. This raises the interesting possibility that β-cleavage of PrP^C^ is an adaptive response, and increased levels of N2 protect the highly sensitive ocular tissues from light-induced oxidative stress. Further exploration is necessary to understand the physiological and pathological implications of this phenomenon fully.

It is remarkable that downregulation of PrP^C^ in TM cells caused significant upregulation of several fibrillogenic proteins including α-SMA, fibronectin, and collagen 1A, suggesting transformation to a mesenchyme-like phenotype^11,13,38,58^. Absence of PrP^C^ in neuronal cells induces aggregation of β1-integrin on the plasma membrane, activating signaling pathways including RhoA-ROCK that interfere with neuronal polarity and axonal growth by altering cell-ECM interactions^5–8,26,59,60^. Our data suggest that a similar mechanism operates in TM cells, and induces upregulation of fibrillogenic proteins typical of the glaucomatous change^12,15^.

PrP^C^ is also a cell surface receptor for laminin, an extracellular matrix glycoprotein that plays a major role in neuronal differentiation^10^. Deletion or dysfunction of PrP^C^ causes aggregation and accumulation of laminin in intra- and extracellular compartments and compensatory upregulation of laminin receptor in astrocytes and neuronal cells^9,10^. Our data show a similar response in TM cells, where upregulation of fibronectin is likely to contribute further to endo-MT-like changes^12,38^. Upregulation of vimentin upon downregulation of PrP^C^ suggests loss of adherens junctions, another characteristic of such a change. Since laminin and vimentin are putative ligands of PrP^C^ ^31^, these changes are likely to be independent of ROCK activation, and suggest that PrP^C^ contributes to Endo-MT-like transition in TM cells by both ROCK-dependent and independent pathways.

Upregulation of myocilin due to silencing of PrP^C^ is difficult to explain from our data. Since exposure to dexamethasone, a known inducer of myocilin did not cause additional upregulation of myocilin in the absence of PrP^C^, it is likely that both pathways intersect, perhaps through ROCK activation^34^. Additional studies are necessary to understand the relationship between PrP^C^ and myocilin.

**Figure 8:**
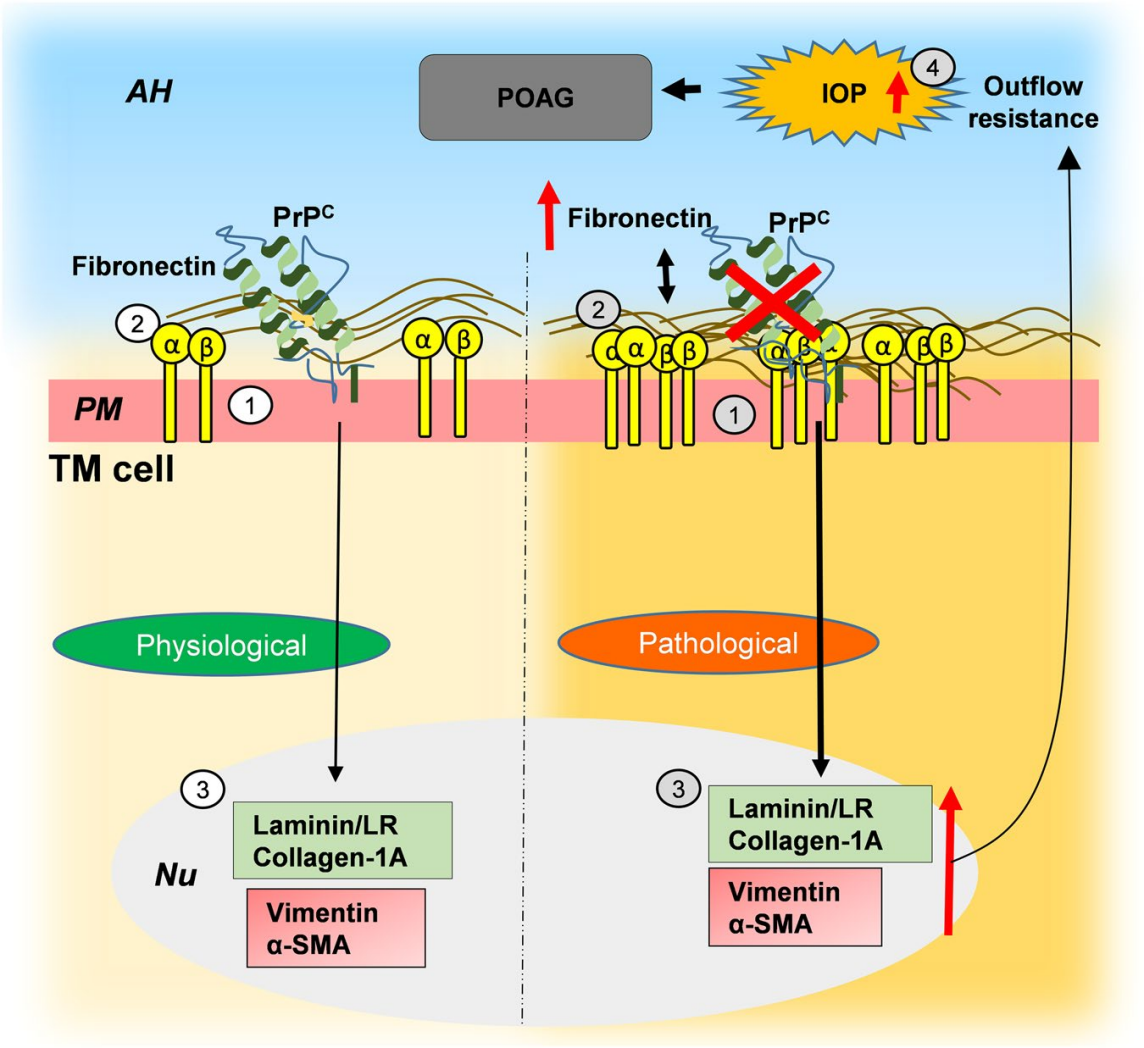
Hypothetical representation of PrP^C^-mediated Endo-MT-like change in the TM. *Physiological role of PrP*^*C*^ *in the TM*: (1) PrP^C^ is expressed on the plasma membrane of TM cells. (2) PrP^C^ maintains cell-ECM interactions by stabilizing β1-integrin and other proteins^5,7^. *Pathological implications*: (1) Downregulation of PrP^C^ induces (2) aggregation of β1-integrin and (3) upregulation of fibronectin, collagen 1A, α-SMA, vimentin, and laminin, resulting in (4) increase in IOP and possibly POAG. AH: aqueous humor; PM: plasma membrane; Nu: nucleus.

In conclusion, this study demonstrates a significant role of PrP^C^ in maintaining cell-ECM interactions TM, and possibly as an anti-oxidant. Downregulation or absence of PrP^C^ induces an endo-MT-like transition in the TM and elevation of IOP in PrP^−/−^ mice, typical of POAG (Fig. 8)^61^. These observations underscore the significance of PrP^C^ as a trigger for endo-TM-like transition in TM cells, and its potential to aggravate glaucomatous pathology due to shedding by ADAM and matrix-metalloproteases in the AH of glaucomatous eyes. Future exploration in additional PrP^−/−^ and over expression mouse models and *ex-vivo* perfusion models of human eye where levels of PrP^C^ have been altered experimentally are necessary to define its precise role in ocular tissues.

## Methods

### Ethics statement

All animal procedures were in accordance with the ARVO Statement for the Use of Animals in Ophthalmic and Vision Research. Animal experiments were approved by the Association for Assessment and Accreditation of Laboratory Animal Care International (AAALAC)-approved Animal Resource Center (ARC) at Case Western Reserve University (CWRU) School of Medicine (SOM).

### Antibodies and Chemicals

HRP-conjugated secondary antibodies (anti-mouse (NA931V), anti-rabbit (NA92V)) were from GE Healthcare (supplied by Sigma, USA). Alexa Fluor 546 (A11071, A11018) and Alexa Fluor 488 (A11017) tagged secondary antibodies were from Southern Biotech, USA and Molecular Probes (supplied by ThermoFisher, USA) respectively. A complete list of antibodies is provided in Table 1. PNGase F (P0704S) was from New England Biolabs (NEB), USA, Lipofectamine 3000 and Lipofectamine RNAiMax were from Invitrogen, USA. Dexamethasone (D1756) was from Sigma Aldrich, USA. siRNA against PrP (sc36318), and scrambled siRNA (sc37007) were from Santa Cruz Biotechnology, USA (sequences provided in Table 2).

**Table 1:** List of antibodies.

Antibody	Host species **	Species reactivity **	Company	Cat.No.	Dilution
PrP (3F4) IgG2a	m	h	Signet laboratories (Dedham, MA)	-	WB-1:250 IHC-1:50
PrP (8H4) IgG2b	m	m, h, b	Sigma Aldrich	P0110	WB-1:250 IHC-1:50
PrP (8B4) IgG_1_	m	m, h	Santa Cruz, USA	sc-47729	WB-1:250
PrP (SAF32) IgG2b	m	m, h, b	Cayman Chemical	189720	WB-1:250 IHC-1:50
PrP (G-12) IgG_1_	m	m, h	Santa Cruz, USA	sc-398451	WB-1:250
PrP (2301) IgG	Rb	h	*Gift by* Dr. Shu Chen, PhD	-	WB-1:500
Laminin receptor IgG	Rb	m, h	Abcam	ab137388	WB-1:1000 IHC-1:50
Alpha smooth muscle actin IgG2a	m	m, Rb, h, b	Abcam	ab7817	WB-1:2000 IHC-1:100
Laminin IgG	Rb	m, Rb, h,	Novus Biologicals	NB300-144	WB-1:1000 IHC-1:50
Fibronectin IgG1	m	h	Santa Cruz, USA	sc59826	WB-1:1000 IHC-1:50
Vimentin IgG1	m	h, b	Santa Cruz, USA	sc32322	WB-1:500 IHC-1:50
Myocilin IgG2b	m	h	Santa Cruz, USA	sc137233	WB-1:500 IHC-1:50
9EG7 anti activated β1 integrin IgG2a	r	m, h	BD Biosciences	553715	ICC- 1:50 WB-1:500
Collagen 1A IgG	Rb	h, b	Rockland antibodies and assays	600-401-103-0.1	WB-1:500 IHC-1:50
β-actin IgG2bκ	m	All species with actin	Millipore, USA	MAB1501	WB-1:5000
Mouse IgG - Control			Abcam	ab37355	IHC: 1:100
Rabbit IgG, Control			Abcam	ab37415	IHC: 1:100

**m: mouse, Rb: rabbit, b: bovine, h: human, r: rat.

**Table 2:** List of biological samples and siRNA used in this study.

*PMI (h)/PCoD*	*Age (year)*	*Gender*	*Tissue region*	
**Human tissues used for primary TM cell culture and whole tissue lysates Source: Lions Gift of Sight**				
14	77	F	Trabecular meshwork/Retina	
4.5	70	F	Trabecular meshwork/Retina/Optic nerve/Ciliary body	
16/Dementia	69	M	Trabecular meshwork/Retina/Optic nerve/Ciliary body	
5.5/ESLD	56	M	Trabecular meshwork/Retina/Optic nerve/Ciliary body	
3/ESRD	78	F	Trabecular meshwork/Retina	
12/Multiple system failure	67	M	Trabecular meshwork/Retina	
7.5/Acute cardiac event	65	M	Trabecular meshwork/Retina	
			TM cells from Dr. Rhee’s lab (from 8 different donors at passage 2).	
**Mouse strain**				
Wild-type and PrP knockout	6 weeks/5M & 5F	C57BL/6	Trabecular meshwork/Anterior Chamber	
**Bovine samples**				
**Breed**			**Tissue region**	
Mixed	—	—	—	Trabecular meshwork/Anterior Chamber
**siRNA**				
**Name**	**Company**	**Cat.No**.	**Sequence**	
Scrambled siRNA	Santa Cruz	sc-37007	Sense: UUCUCCGAACGUGUCACGUtt Antisense: ACGUGACACGUUCGGAGAAtt	
PrP siRNA (h) is a pool of 3 different siRNA duplexes	Santa Cruz	sc-36318	sc-36318A: Sense: GUGACUAUGAGGACCGUUAtt Antisense: UAACGGUCCUCAUAGUCACtt sc-36318B: Sense: GAGACCGACGUUAAGAUGAtt Antisense: UCAUCUUAACGUCGGUCUCtt sc-36318C: Sense: GUUGAGCAGAUGUGUAUCAtt Antisense: UGAUACACAUCUGCUCAACtt	

### Culture and characterization of human TM cells

Primary cultures of human TM cells were obtained from the Rhee laboratory and established from eye globes using the standard protocol^35,62^, and characterized before by checking upregulation of myocilin in response to dexamethasone^35^ (Supplementary Fig. S1a-b). Primary human TM cell cultures were derived from several donors (age range: 56–78). After ciliary body (CB) was removed using scalpel, a cut in the rim of TM was made and the TM tissue was pulled out carefully using surgical grade forceps. Then, we made six sections of TM tissue using scalpel. Tissue sections were washed once in clean media and placed into the well of 6- wellplates. A coverslip was washed twice in clean media and placed gently over tissue sample keeping tissue sample toward center of well. Air bubbles were avoided during the whole process. The well was carefully handled in order not to scratch bottom of wells. Three milliliter of the media were added to each well dropping directly over the coverslip. The plate was incubated in 37 °C, 10% of CO_2_ incubator. The growth of TM was checked for 3 weeks. Once the cells grew, media was replaced twice a week until cells grew confluent. TM cells were maintained in the growth medium consisted of Dulbecco’s modified Eagle’s medium (DMEM) supplemented with 20% fetal bovine serum (FBS) (Invitrogen-Gibco, Grand Island, NY), 1% L-glutamine (2 mM), and 0.5% or 0.1% gentamicin (50 or 10 µg/mL). For further experiment, TM cultures were seeded into 6-well plates, allowed to grow to confluence at 37 °C in a 10% CO_2_ atmosphere, and given an additional 2–3 days for differentiation. Confluent cultures of TM cells were used for all biochemical studies. For immunocytochemistry sub-confluent cultures were used to facilitate visualization of the plasma membrane. Both confluent and sub-confluent cultures responded to dexamethasone by upregulating myocilin (Supplementary Fig. S1c). To silence PrP^C^, the cells were transfected with PrP-specific or the corresponding scrambled siRNA using Lipofectamine RNAiMax as per manufacturer’s instructions. Desired downregulation of PrP^C^ was confirmed by Western blotting.

### Human and bovine eye samples

Human eye globes were acquired from Lions Gift of Sight eye bank (1000 Westgate Dr Ste 260 Saint Paul, MN 55114). The donors ranged in age from 42–78 years. Other available details of donors are provided in Table 2. Bovine eyes were collected from a local abattoir. The samples were either fixed in buffered formalin (1:10) for immunohistochemistry, or dissected to isolate the desired tissues.

### Mouse strains

PrP-knock out (PrP^−/−^) mice were obtained from Jackson Laboratories (cat # 129-Prnptm2Edin/J Stock No: 012938) and crossed with C57BL/6 wild-type mice for 10 generations. F2 generation of wild type (PrP^+/+^) and corresponding PrP^−/−^ (PrP^−/−^) were used for these studies. Control and experimental mice were ~6 weeks old, sex matched, and maintained under similar conditions.

### Tissue preparation and Immunohistochemistry

Immunocytochemistry and immunohistochemistry were performed as described^43^. In short, thin sections of formalin-fixed TM tissue or primary human TM cells cultured on coverslips were processed for immunoreaction with the desired primary antibody followed by Alexa Fluor-conjugated secondary antibody. The nuclei were stained with Hoechst (#33342, Invitrogen, USA). Stained specimens were mounted and imaged with Leica inverted microscope (DMi8). Each experiment was repeated 3–4 times, and a representative image from 10 different fields is shown. Images of control sections reacted with isotype specific irrelevant primary antibody or buffer are shown in respective figures. Additional care was taken to identify the trabecular meshwork area for all sections in all the 3 species and the H&E staining for all sections analyzed were carried out and provided in the respective figures.

### SDS-PAGE and Western blotting

Protein lysates and aqueous humor were fractionated by SDS-PAGE and analyzed by Western blotting as described^30^. For Collagen1A blotting, the samples were processed in a non-reducing and non-denaturing condition. Quantification of protein bands was performed by densitometry using UN-SCAN-IT gels (version6.1) software (Silk Scientific, USA) and ImageJ Software analyzed graphically using GraphPad Prism (Version 5.0) software (GraphPad Software Inc., USA) and Microsoft excel. Full-length blots are included in Supplementary Fig. S2.

### IOP measurement

IOP was measured at the same time of day with TonoLab tonometer (Colonial Medical Supply; USA-Icare, Finland). Mice were anesthetized with ketamine/xylazine before the measurement, and six measurements were obtained for each eye per animal. Average of all values was used for analysis.

### Statistical analysis

Quantification of protein bands was performed and presented as Mean ± SEM of the indicated n. Level of significance was calculated by Two-way ANOVA between the control and experimental groups.

## Supplementary information

Supplementary info
