# Supplementary material for: Prion protein modulates endothelial to mesenchyme-like transition in trabecular meshwork cells: Implications for primary open angle glaucoma
Source: Sci Rep. 2019 Sep 11;9:13090. doi: 10.1038/s41598-019-49482-6 (PMC6739364; doi:10.1038/s41598-019-49482-6)
Supplement: Supplementary file 1 — Supplementary info [file 41598_2019_49482_MOESM1_ESM.docx]

**Supplementary Data**

**Prion protein modulates endothelial to mesenchyme-like transition in trabecular meshwork cells: Implications for primary open angle glaucoma**

**Ajay Ashok^1^, Min H Kang^2^, Aaron S Wise^1^, Pattabiraman P^2^, William M Johnson^3^, Michael Lonigro^1^, Ranjana Ravikumar^1^, Douglas J Rhee^2^, Neena Singh^1^***

***Corresponding author**

**E-mail:** [**neena.singh@case.edu**](mailto:neena.singh@case.edu)

**Tel: 216-368-2617**

**Grant information**: Funded by R01 NS 092145 to NS

**Departments of Pathology^1^ and Ophthalmology^2^, School of Medicine, Case Western Reserve University, Cleveland, Ohio 44106.**

**Supplementary Figure 1 (S1)**

**Human trabecular meshwork (huTM) cells characterization**

Human TM cells were isolated and characterized using Dexamethasone (Dex) treatment and estimating myocilin expression. Cell lysates from control and Dex treated cells were subjected to Western blotting and it was observed that myocilin expression increased significantly following Dex treatment which confirms the purity of the cells isolated **(Fig S1 a,b)**. Furthermore, to analyze the difference between confluent and sub confluent cells, huTM cells were stained with myocilin to observe any change in cellular characteristics. There was no significant change in myocilin expression between confluent and sub-confluent cells including cell morphology **(Fig S1c)**.

**
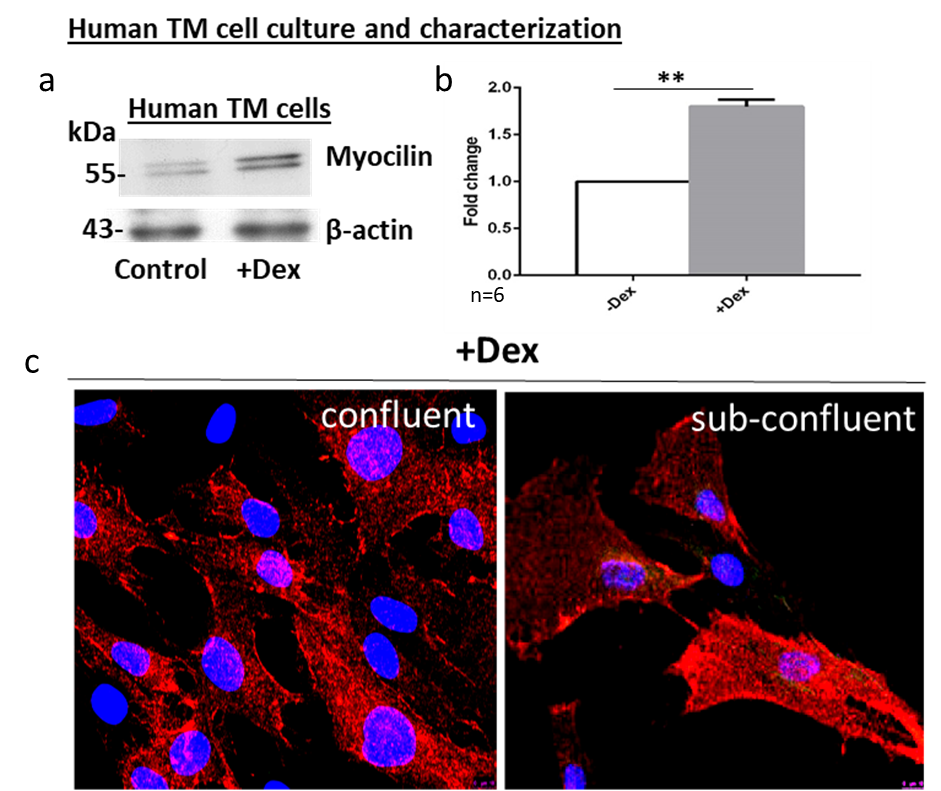
**

**Myocilin**/**Nuclei**

**Legend: Fig. S1. a)** Characterization of primary huTM cells were carried out by evaluation of cell lysates from control and Dex treated cells by Western blotting and probing with myocilin. Myocilin expression increased in the Dex treated cells thereby confirming the purity of huTM cells. Full blots presented in Supplementary Figure S2. **b)** Quantification of myocilin expression by densitometry after normalization with β-actin shows significant upregulation following Dex treatment. Values are mean ± SEM of the indicated n. ***p*<0.01. **c)** ICC staining of confluent and sub-confluent huTM cells following probing with myocilin. Cells did not show any significant changes in morphology and protein expression thereby confirming that the characteristics of cells don’t alter when cultured at subconfluency.

**Full blot for Fig. S1**

**
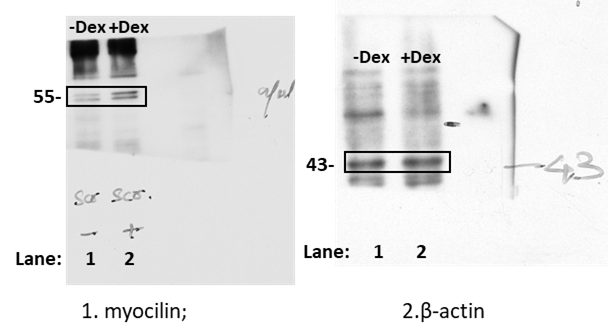
**

**Supplementary Figure 2 (S2)**

**Fig S2.** Full blots for all cropped western blots are presented.

**Fig. 3 in manuscript**


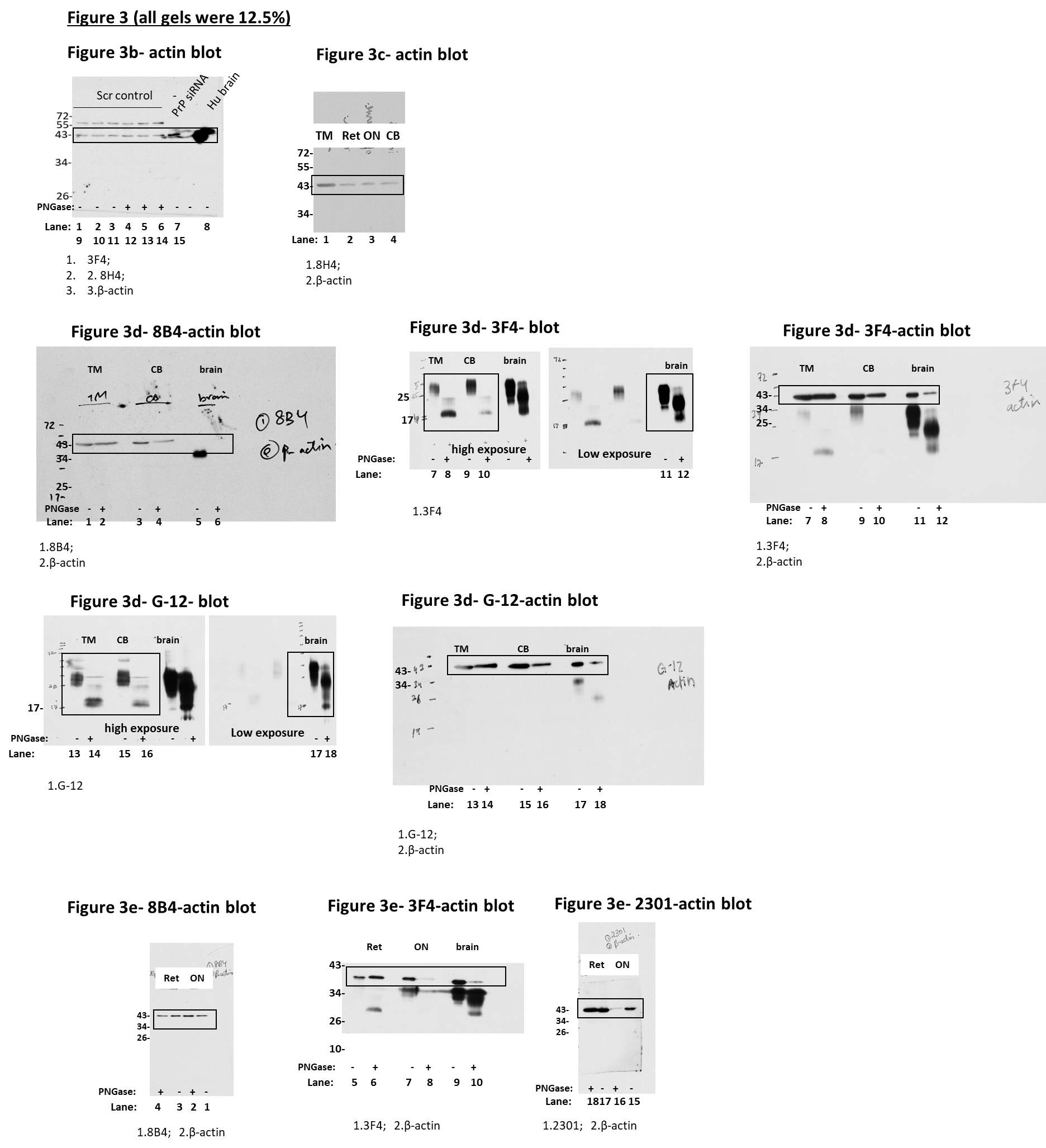


**Legend:** Full blots for Fig. 3.

**Fig. 4 in manuscript**

**
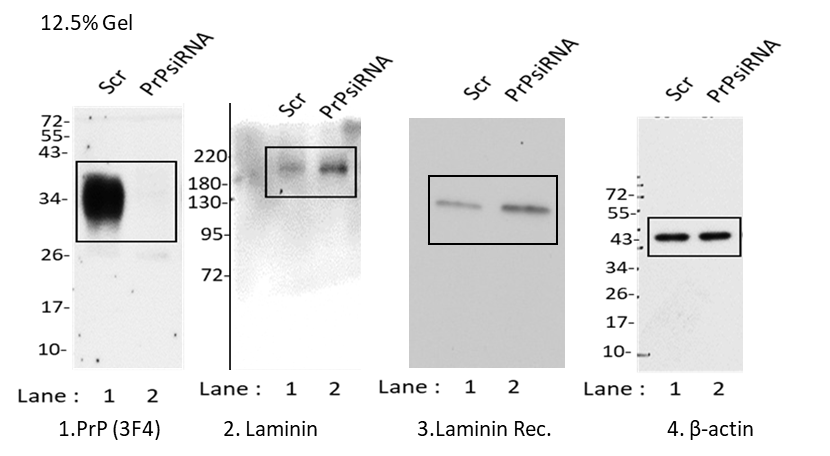
**

**Legend:** Full blots for Fig. 4.

**Fig. 5 in manuscript**


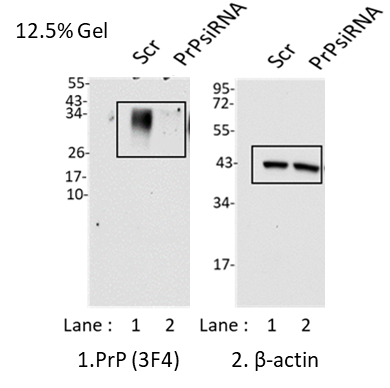

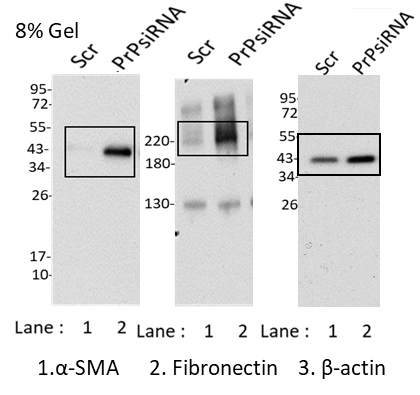


**Legend:** Full blots for Fig. 5.

**Fig. 6 in manuscript**


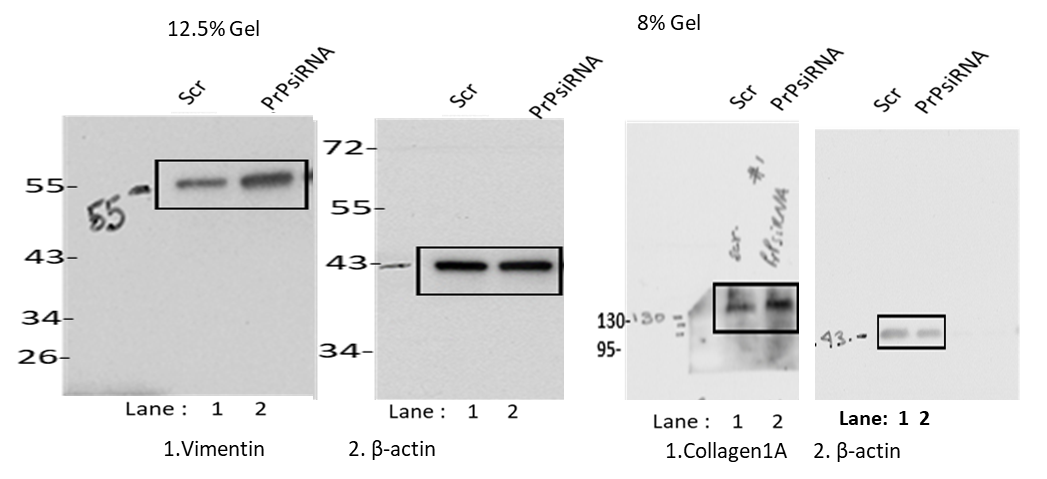


**Legend:** Full blots for Fig. 6.

**Fig. 7 in manuscript**


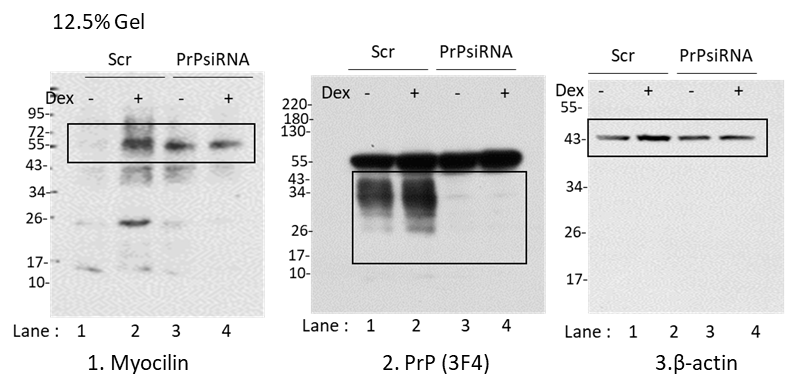


**Legend:** Full blots for Fig. 7.

**Supplementary Fig. 3 (S3)**

**Prion processing in TM and CB**

Human TM and CB lysates were processed and probed with 2301 which validated the G-12 probing results in Figure 3 in the manuscript. TM undergoes PrP^C^ processing and produces both C1 and C2 fragments. However, CB deglycosylation yields more C1 (18kDa) fragment relative to the C1 form.


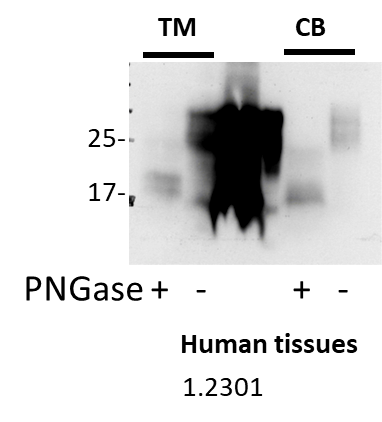


**Legend: Fig. S3.** Lysates from human TM and CB were deglycosylated and probed with 2301 antibody to identify different forms of PrP^C^. The data validated the results obtained with G-12 antibody provided in Figure 3d.

**Supplementary Fig. 4 (S4)**

No significant difference was observed in β1 integrin expression in human TM cells transfected with PrP-siRNA relative to controls (lane 1 and 2).


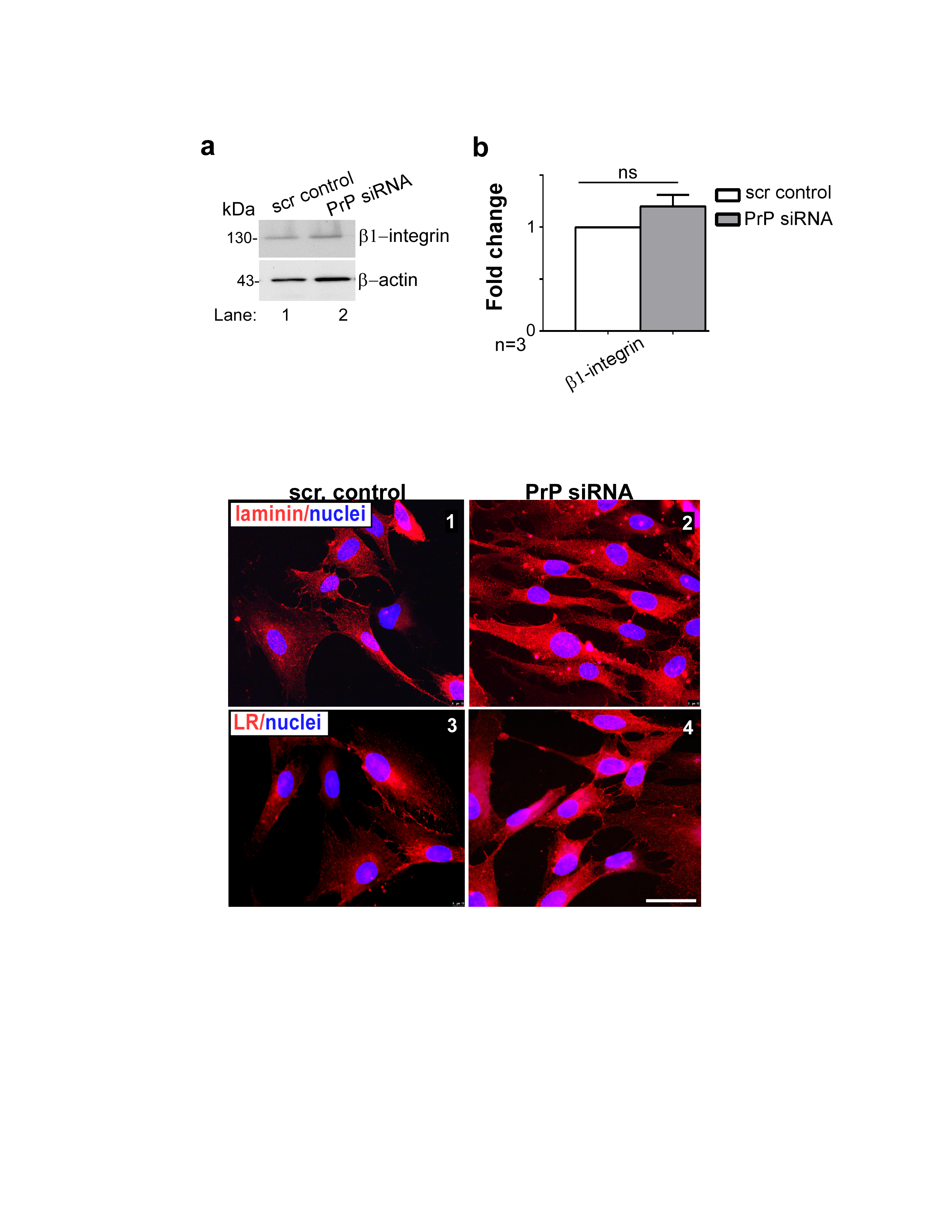


**Legend: Fig. S4. (a & b)** PrP^C^ was silenced in human TM cells as above, and lysates were processed for Western blotting. Probing for β1 integrin shows no significant change in the absence of PrP^C^ relative to controls (lanes 1 & 2). Values are mean ± SEM of the indicated n.

**Full blot for Fig. S4**

**
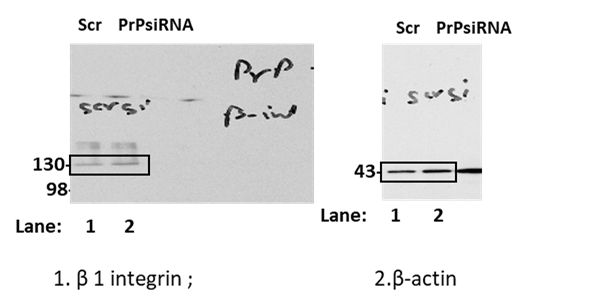
**

**Supplementary Fig. 5 (S5)**

Immunostaining of cells, showed stronger reactivity for laminin and laminin receptor in cells transfected with PrP-siRNA relative to controls (panels 1-4).


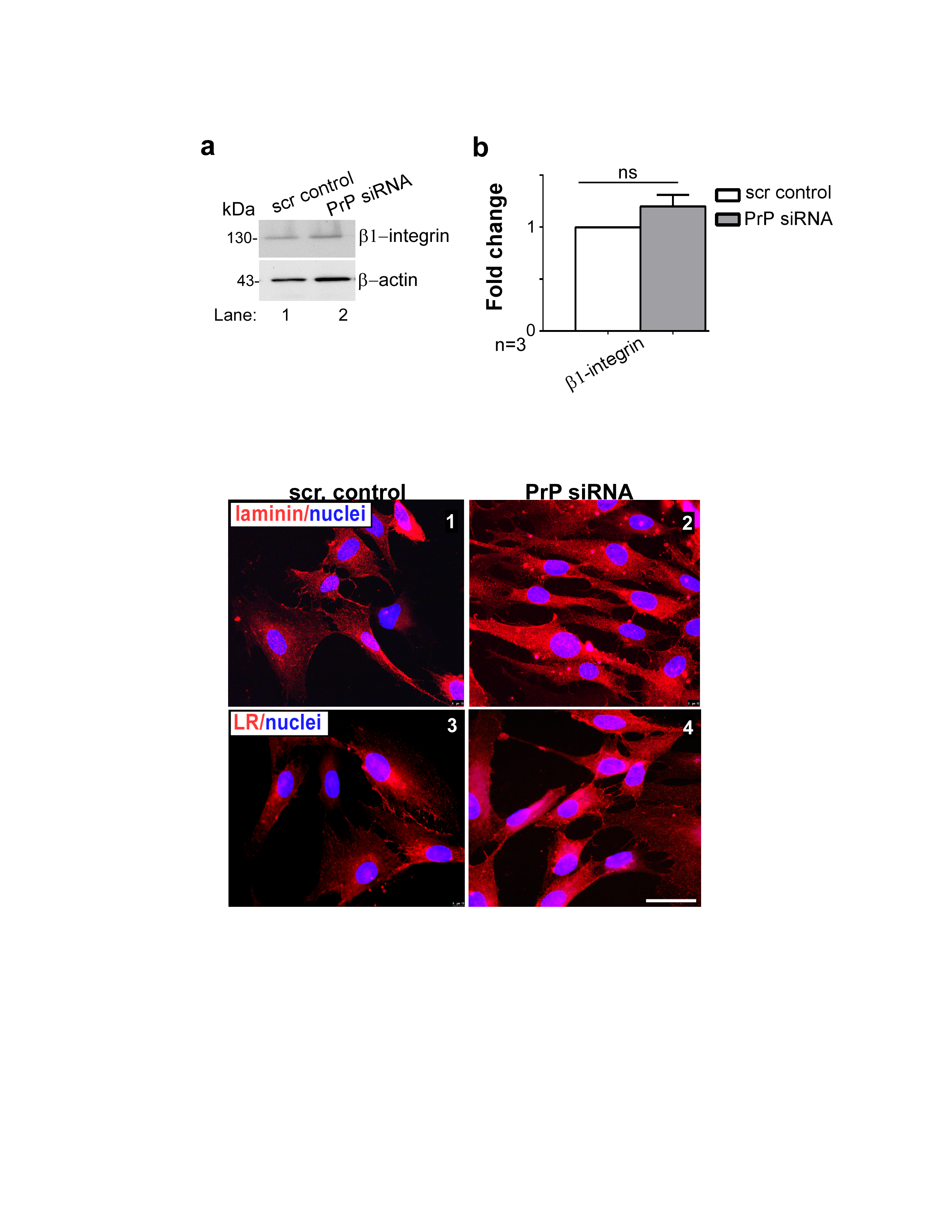

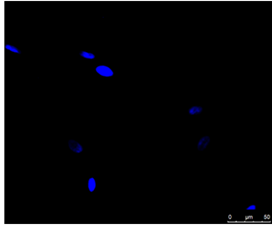


Rabbit IgG control 5

**Legend: Fig. S5.** Immunostaining for laminin shows cytoplasmic and extracellular punctate reaction in the absence of PrP^C^ (panels 1 & 2). Reactivity for laminin receptor is increased upon downregulation of PrP^C^ (panels 3 & 4). Scale bar: 25µm. No reaction was detected in the IgG control processed in parallel (panel 5).
